# Avoiding misdiagnosis: expert consensus recommendations for the suspicion and diagnosis of transthyretin amyloidosis for the general practitioner

**DOI:** 10.1186/s12875-020-01252-4

**Published:** 2020-09-23

**Authors:** Morie Gertz, David Adams, Yukio Ando, João Melo Beirão, Sabahat Bokhari, Teresa Coelho, Raymond L. Comenzo, Thibaud Damy, Sharmila Dorbala, Brian M. Drachman, Marianna Fontana, Julian D. Gillmore, Martha Grogan, Philip N. Hawkins, Isabelle Lousada, Arnt V. Kristen, Frederick L. Ruberg, Ole B. Suhr, Mathew S. Maurer, Jose Nativi-Nicolau, Candida Cristina Quarta, Claudio Rapezzi, Ronald Witteles, Giampaolo Merlini

**Affiliations:** 1 Mayo Clinic, 200 First Street SW, Rochester, MN 55905 USA; 2 Referral Center for FAP, Neurology Department, APHP, INSERM U 1195, Université Paris-Sud, Le Kremlin Bicêtre, France; 3 Department of Neurology, Graduate School of Medical Sciences, Kumamoto University, Kumamoto, Japan; 4 Ophthalmology Service, Hospital de Santo António, Porto, Portugal; 5 Columbia University Medical Center, New York, NY USA; 6 Centro Hospitalar do Porto, Porto, Portugal; 7 John C. Davis Myeloma and Amyloid Program, Tufts Medical Center, Boston, MA USA; 8 Department of Cardiology, Referral Center for Cardiac Amyloidosis, GRC Amyloid Research Institute, DHU A-TVB, APHP CHU Henri Mondor and Université Paris Est Créteil, Créteil, France; 9 Brigham and Women’s Hospital, Boston, MA USA; 10 University of Pennsylvania Perelman School of Medicine, Philadelphia, PA USA; 11 National Amyloidosis Centre, University College London, London, UK; 12 Amyloidosis Research Consortium, Newton, MA USA; 13 University of Heidelberg, Heidelberg, Germany; 14 Boston University School of Medicine, Boston Medical Center, Boston, MA USA; 15 Department of Public Health and Clinical Medicine, Umeå University, Umeå, Sweden; 16 University of Utah Health, Salt Lake City, UT USA; 17 University of Bologna, Bologna, Italy; 18 Stanford Amyloid Center, Stanford University School of Medicine, Stanford, California, USA; 19 Amyloidosis Research and Treatment Center Foundation, IRCCS Policlinico San Matteo, San Matteo, Italy; 20 Department of Molecular Medicine, University of Pavia, Pavia, Italy

**Keywords:** ATTR amyloidosis, ATTRv, Diagnosis, hATTR, Polyneuropathy, Cardiomyopathy, Transthyretin amyloidosis

## Abstract

**Background:**

Transthyretin amyloidosis (also known as ATTR amyloidosis) is a systemic, life-threatening disease characterized by transthyretin (TTR) fibril deposition in organs and tissue. A definitive diagnosis of ATTR amyloidosis is often a challenge, in large part because of its heterogeneous presentation. Although ATTR amyloidosis was previously considered untreatable, disease-modifying therapies for the treatment of this disease have recently become available. This article aims to raise awareness of the initial symptoms of ATTR amyloidosis among general practitioners to facilitate identification of a patient with suspicious signs and symptoms.

**Methods:**

These consensus recommendations for the suspicion and diagnosis of ATTR amyloidosis were developed through a series of development and review cycles by an international working group comprising key amyloidosis specialists. This working group met to discuss the barriers to early and accurate diagnosis of ATTR amyloidosis and develop a consensus recommendation through a thorough search of the literature performed using PubMed Central.

**Results:**

The cardiac and peripheral nervous systems are most frequently involved in ATTR amyloidosis; however, many patients often also experience gastrointestinal and other systemic manifestations. Given the multisystemic nature of symptoms, ATTR amyloidosis is often misdiagnosed as a more common disorder, leading to significant delays in the initiation of treatment. Although histologic evaluation has been the gold standard to confirm ATTR amyloidosis, a range of tools are available that can facilitate early and accurate diagnosis. Of importance, genetic testing should be considered early in the evaluation of a patient with unexplained peripheral neuropathy.

**Conclusions:**

A diagnostic algorithm based on initial red flag symptoms and manifestations of cardiac or neurologic involvement will facilitate identification by the general practitioner of a patient with clinically suspicious symptoms, enabling subsequent referral of the patient to a multidisciplinary specialized medical center.

## Background

Transthyretin amyloidosis (also known as ATTR amyloidosis) is a devastating, life-threatening, and underrecognized disease wherein misfolded transthyretin (TTR) protein forms fibrils and deposits in organs and tissue, disrupting normal organ function and tissue structure [1–3]. Under homeostatic conditions, tetrameric TTR complexes transport thyroid hormone and retinol binding protein [4, 5]. With ATTR amyloidosis, amyloid fibril formation occurs because of a single amino acid substitution or complex instability, causing tetrameric TTR to dissociate into monomers, which misfold and aggregate before depositing into tissue and organs [4, 6]. Accumulation of TTR amyloid fibrils results in multisystem dysfunction with clinical manifestations observed in the heart, musculoskeletal system, peripheral nervous system, and autonomic nervous system. ATTR amyloidosis is a progressive and systemic disease and may be either hereditary (ATTRv; v for variant) or sporadic (ATTRwt; wt for wild type) [7]. ATTRv and ATTRwt amyloidosis can result in a heterogeneous, multisystem presentation of clinical manifestations in patients with life expectancy depending on several factors, such as the form of disease, age, predominant phenotype, comorbidities, and duration of symptoms before diagnosis (Table 1). Although ATTR amyloidosis was previously untreatable, the landscape changed quickly in 2018 to 2019 with the US Food and Drug Administration approval of three new, effective disease-modifying therapies for the treatment of ATTRv-related peripheral neuropathy (ATTRv-PN) and ATTRv or ATTRwt-related cardiomyopathy (ATTRv-CM or ATTRwt-CM, respectively) [29–31].

**Table 1:** Clinical manifestations of ATTRv and ATTRwt amyloidosis

	ATTRv	ATTRwt	References
Age at symptom onset	> 20 years	> 50 years	[8–12]
Male, %	76–86	91–97	[13–15]
Duration of symptoms before diagnosis	~ 3 years	~ 2 years	[8, 9, 11]
Median life expectancy, after diagnosis	• 2–5 years with predominantly CM • 8–10 years with predominantly PN	4 years	[9, 12, 16, 17]
Clinical manifestation			
Cardiac	Yes	Yes	[13, 18, 19]
Peripheral nerves	Yes	Occasionally	[13, 18]
Autonomic nerves (including gastrointestinal)	Yes	Rare	[13, 18, 20, 21]
Kidney	Yes	Rare	[1, 13]
Ophthalmologic	Vitreous deposition	Not prominent	[1]
Musculoskeletal	Yes	Yes	[13, 22–28]

*ATTRv* Hereditary ATTR amyloidosis, *ATTRwt* Wild-type ATTR amyloidosis, *CM* Cardiomyopathy, *PN* Polyneuropathy

The prevalence of ATTR amyloidosis is higher than previously recognized. An autopsy study found that ATTRwt may be present in as many as 25% of persons older than 80 years [32], whereas a separate autopsy study noted that ATTRwt may be the cause of heart failure (HF) in 5% of patients with HF with preserved ejection fraction (HFpEF) [33]. Nuclear scintigraphy has suggested that 13% of patients admitted to the hospital with HFpEF and as many as 16% of elderly patients with severe degenerative aortic stenosis may have ATTRwt-CM [34, 35]. One genetic variant, *V122I*, is carried by 3.4% of African Americans. This means that approximately 1.5 million people (out of 50 million total African Americans in 2018) are carriers of and are therefore at elevated risk for cardiac amyloidosis [36–40]. The genetic variants causing ATTRv follow an autosomal dominant pattern; consequently, ATTRv has typically been considered an endemic disease with an early onset and is prevalent in endemic regions (such as Japan, Sweden, and Portugal) [16, 41]. However, penetrance is variable and has not been assessed for many variants [42]. ATTRv-PN is now considered to be a worldwide disease, with a global prevalence estimated at 10,186 cases [43].

The aim of this article is to heighten disease awareness, to increase clinical suspicion in the presence of signs and symptoms suggestive of ATTR amyloidosis, and to promote proper testing and referrals among general practitioners, who are typically the first point of contact for a newly symptomatic patient. Moreover, symptoms are often heterogeneous and can be incorrectly associated with a more common disorder.

## Methods

These consensus recommendations for the suspicion and diagnosis of hereditary and wild-type ATTR amyloidosis were developed through a series of development and review cycles through in-person meetings, along with refinement of the draft by telephone or email. The Amyloidosis Research Consortium has created a collaborative research model to bring together experts in the amyloidosis field to address the challenges that exist in developing diagnostic tools and carrying out innovative clinical trials. An international working group composed of key amyloidosis specialists in collaboration with companies conducting research in ATTR amyloidosis (GlaxoSmithKline, Ionis, Pfizer, and Alnylam) and the Amyloidosis Research Consortium was assembled. The international working group met to discuss challenges to early and accurate diagnosis of ATTR amyloidosis and to develop a consensus recommendation for the diagnosis of ATTR amyloidosis, recognizing that whether a patient presents in an endemic or a non-endemic country and has early or late-onset disease will influence the patient pathway to diagnosis. An initial thorough search of the literature was performed using PubMed Central and the following search terms: (amyloid OR amyloidosis) AND transthyretin OR (senile or SSA or SCA or TTR or ATTR or cardiac or FAP or FAC or mutant or hereditary or familial or neuropathy or ATTRv or mATTR or hATTR or wtATTR). The results of this broad and expansive search were further refined focusing on references concerning suspicion of disease and diagnosis, and the resultant list was consequently reviewed by the working group. Although only one database was searched, the initial search was exhaustive and the review by the authors, who are global experts in the field, ensured that all the appropriate references were identified. Several publications have sought to provide clinical guidelines for the diagnosis and management of ATTR amyloidosis [1, 16, 41, 44, 45]. Five clinical practice guidelines were analyzed to assess similarities and differences between them [1, 16, 41, 44, 45]; these findings were incorporated, along with the results of the literature search and the medical expertise of the international working group, to develop the final consensus recommendations.

## Results

### Underrecognition

**Fig. 1:**
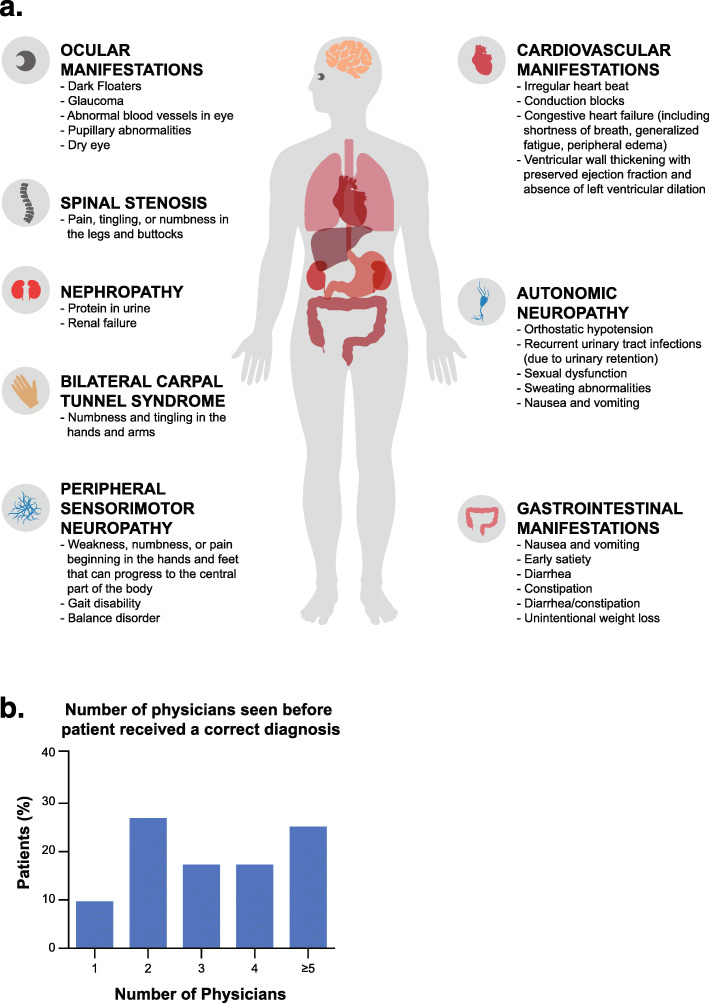
Systemic manifestations of ATTR amyloidosis commonly result in delayed diagnosis. **a** The varied systemic manifestations of ATTR amyloidosis. Modified with permission from Conceição I, et al. [1]. **b** The number of physicians seen before a patient is correctly diagnosed. Adapted with permission from Lousada L, et al. [46]. ATTR, transthyretin amyloid

Although prevalence is higher than previously recognized, ATTR amyloidosis remains a challenging disease to identify largely because it manifests with varied symptoms and may involve multiple organs (Fig. 1a) [1]. Symptoms often mimic those of other more common diseases, rendering accurate diagnosis of ATTR amyloidosis difficult [46, 47]. In a survey of patients with ATTR amyloidosis, diagnosis was made within 6 months in only 35% of those with ATTRv and 46% of those with ATTRwt, with many patients seeing more than five physicians before receiving a correct diagnosis (Fig. 1b) [46, 47].

The cardiac and peripheral nervous systems are most commonly involved in patients with ATTR amyloidosis. ATTR-CM (transthyretin amyloid cardiomyopathy) typically causes heart failure and/or arrhythmias, whereas neurologic involvement is characterized by a progressive peripheral and autonomic neuropathy that can rapidly become severe and disabling. Early identification and intervention are crucial to improve patient outcomes because newly available treatments have been shown to have maximum therapeutic benefit when started in the early stages of the disease.

In recent years, contemporary cardiac imaging techniques, including magnetic resonance imaging (MRI) and bone scintigraphy, have dramatically altered the diagnostic algorithm for ATTR-CM, which has resulted in increased detection, demonstrated by an increase in patient referrals to the National Amyloidosis Centre in London in the United Kingdom since 2012 [47]. These referred patients, however, have still routinely presented with advanced HF and poor quality of life [47]. In addition to increasing disease awareness among physicians, screening algorithms that capture patients with known ATTR variants or family members of patients with known disease can help identify affected but clinically unrecognized individuals. In addition, emerging data indicate that ATTR-CM screening should be considered for older adults with bilateral carpal tunnel syndrome (CTS) [22], spinal stenosis [23, 48], HFpEF, and/or a hypertrophic cardiomyopathy phenotype to improve disease detection and diagnosis [34]. Earlier diagnosis is crucial for all patients irrespective of genotype, and, given the inherited nature of ATTRv, diagnosis of one patient with ATTRv has important ramifications for all first-degree relatives, who may then be monitored and treated as soon as symptoms arise [49].

### Burden of disease

The burden of disease is high for patients and caregivers. Patients who completed the 36-Item Short Form Health Survey (SF-36) reported scores up to 2 standard deviations below those seen in the general population for physical health, quality of life, and work productivity. Neuropathy-specific quality of life for patients with ATTRv, as measured with the Norfolk QOL-Diabetic Neuropathy questionnaire, was nearly equivalent to that of patients with type 2 diabetes with diabetic neuropathy accompanied by a history of ulceration, gangrene, or amputation. Generic quality of life, as measured with the SF-36, was worse than that seen in the general population, and physical functioning was worse than that for patients with multiple sclerosis and congestive HF [50]. Caregivers also report poor mental health, poor work productivity, and a considerable amount of time required to provide care (mean, 45.9 h/week) [51]. It has also been shown that patient quality of life is poor at the time of diagnosis, indicating that substantial ATTR amyloidosis disease progression has often occurred before that time [47].

## Discussion

The diagnostic process leading to the final diagnosis of ATTR amyloidosis can be framed in two distinct (albeit interconnected) phases: clinical suspicion and diagnosis. Both phases have many pitfalls, and the steps involved can be challenging.

### Clinical suspicion of cardiac involvement: presenting symptoms and common misdiagnoses

The most common presenting signs and symptoms of ATTR-CM are orthopnea, paroxysmal nocturnal dyspnea, fatigue, exercise intolerance, dizziness/syncope, palpitations, atrial fibrillation, thromboembolism, and fluid retention [13, 34, 52–55]. Cardiac symptoms are sometimes accompanied by other systemic manifestations, such as unexplained peripheral neuropathy, or gastrointestinal (GI) symptoms, such as early satiety, nausea, vomiting, and/or altered bowel habits [20]. A history of surgically corrected bilateral CTS and/or lumbar stenosis is a red flag and should prompt consideration of ATTR amyloidosis because these symptoms can be early manifestations of the disease (ATTRwt and ATTRv) [22–27].

Echocardiographic (ECHO) findings of ATTR amyloidosis can often lead to a misdiagnosis of hypertrophic cardiomyopathy; in older adults, ATTR amyloidosis is the most common phenocopy for hypertrophic cardiomyopathy [56]. Hypertensive cardiac remodeling and undifferentiated HFpEF are also common misdiagnoses (Table 2) [34].

**Table 2:** Common Misdiagnoses of Disturbances Caused by ATTR Amyloidosis

Common Misdiagnosis	ATTR Symptoms Contradicting Given Diagnosis
**Cardiac**	
Hypertrophic cardiomyopathy	Discordant voltage to mass ratio
Hypertensive heart disease	Discordant voltage to mass ratio; intolerance to beta blockers; waning need for antihypertensives
Undifferentiated heart failure with preserved ejection fraction	Nondilated hypertrophic LV
Uncomplicated degenerative aortic stenosis	Reduced longitudinal strain Frequent low-flow, low-gradient paradoxical pattern Thickened atrioventricular valves
**Neurologic**	
Chronic inflammatory demyelinating polyneuropathy	Pain in the limbs, dysautonomia (erectile dysfunction, OH), symmetric polyneuropathy in upper limbs
Monoclonal gammopathy–associated neuropathy	Autonomic dysfunction (erectile dysfunction, OH)
Idiopathic axonal polyneuropathy	Dysautonomia (erectile dysfunction, OH), walking difficulties
CTS	Worsening of upper limb symptoms despite CTS surgery
Lumbar spinal stenosis	Failure to relieve symptoms in spite of spine surgery
Diabetic neuropathy	Walking difficulties
Amyotrophic lateral sclerosis	No upper motor neuron syndrome Reduction of amplitude of SNAP
Motor neuropathy	Reduction of amplitude of SNAP
**Gastrointestinal**	
Inflammatory bowel syndrome	Absence of inflammation
Irritable bowel syndrome	Absence of or only minor abdominal pain; weight loss
Idiopathic diarrhea Idiopathic bile acid malabsorption	Weight loss
Pseudo-obstruction	Absence of or only minor abdominal pain or radiologic findings of intestinal obstruction

*ATTR* Transthyretin amyloidosis, *CTS* Carpal tunnel syndrome, *GI* Gastrointestinal, *LV* Left ventricle, *OH* Orthostatic hypotension, *SNAP* Sensory nerve action potential

**Fig. 2:**
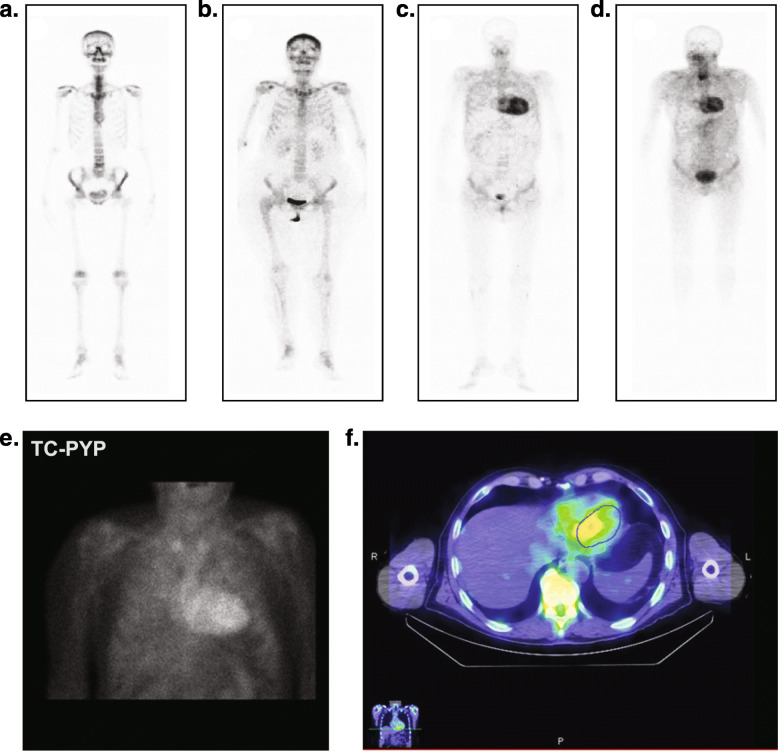
Assessments for noninvasive diagnosis of ATTR amyloidosis. (A-D) ^99m^Tc-DPD bone tracer scintigraphy. **a** No uptake outside of bone (score 0) is typical of patients without ATTR amyloidosis. **b** Some uptake outside of bone without myocardial uptake (score 1) may be seen in AL amyloidosis or possibly ATTR amyloidosis; if suspicion is high, consider a biopsy. **c** Moderate (score 2, myocardial uptake = rib uptake) and **d** high (score 3, myocardial uptake > rib uptake) uptake in the heart along with suspicious symptoms is diagnostic for ATTR amyloidosis (serum and urine immunofixation and FLC levels must be normal to discount AL amyloidosis). **e**, **f** ^99m^Tc-PYP bone tracer scintigraphy. **e** Planar chest and **f** SPECT chest scans that demonstrate uptake both in blood pool and in the myocardial wall. ^99m^Tc-DPD, technetium-99 m-3,3-diphosphono-1,2 propanodicarboxylic acid; ^99m^Tc-PYP, technetium-99 m pyrophosphate; AL, light-chain amyloidosis; ATTR, transthyretin amyloid; FLC, free light chain; SPECT, single photon emission computed tomography. **a**-**d** Reused with permission from Perugini E, et al. [60]. **e**-**f** Courtesy of Morie Gertz

Further signs of amyloidosis are seen on ECHO or cardiac magnetic resonance (CMR) imaging; specifically, left ventricular walls are nearly invariably thickened, and coexisting pericardial effusion, right ventricular wall thickening, and interatrial septal thickening are often seen [57]. Another important finding is a disproportion between left ventricular wall thickness and QRS voltages [58, 59]. Readily available tests that can assist in raising the index of clinical suspicion include ECHO, electrocardiography (ECG), CMR with late gadolinium enhancement/extracellular volume, or bone scintigraphy (technetium-99 m-labeled pyrophosphate [PYP], 3,3-diphosphono-1,2-propanodicarboxylic acid [DPD], hydroxymethylene diphosphonate [HMDP]) (Fig. 2, Table 3) [53, 60–64]. Use of serum cardiac biomarkers such as troponin levels, N-terminal probrain natriuretic peptide plasma levels, and free light chain/serum and urine immunofixation electrophoresis to exclude light-chain amyloidosis (AL) are also diagnostically useful [53, 63, 64].

**Table 3:** Clinical Tests and Findings Potentially Suggestive of ATTR Amyloidosis

**Heart**	
ECG	Normal or low ECG voltage^a^ often discrepant from ECHO findings, pseudo-infarct pattern, atrioventricular block, bundle branch block
ECHO	Increased left and/or right ventricular wall thickness, increased atrial septal thickness, impaired longitudinal strain, apical sparing pattern by longitudinal strain, thickened valve leaflets, increased LV filling pressures, pericardial effusion
CMR	Increased biventricular wall thickness, increased LV mass, diffuse subendocardial or transmural late gadolinium enhancement, increased native noncontrast T1 and ECV
^99m^Tc bone scintigraphy (DPD/PYP/HMDP)	Grade 2/3 myocardial uptake; note, this test should always be ordered with serum FLC/serum and urine immunofixation electrophoresis to rule out the presence of a monoclonal protein. If any of these are abnormal, endomyocardial biopsy with typing of amyloid fibril may be necessary for an accurate diagnosis
Serum cardiac biomarkers	Increased BNP or NT-proBNP levels, increased troponin T or troponin I levels
**Peripheral nerves**	
Nerve conduction study	Axonal sensorimotor neuropathy, CTS
Neuro MRI	Swelling of dorsal ganglia
**Autonomic nerves**	
Schellong test	Neurologic orthostatic hypotension
CVRR	Decreased CVRR
Sweat test Laser Doppler flowmetry	Anhidrosis, hypohidrosis

*ATTR* Transthyretin amyloid, *ATTRwt* Wild-type transthyretin amyloidosis, *BNP* Brain natriuretic peptide, *CMR* Cardiovascular magnetic resonance, *CT* Computed tomography, *CTS* Carpal tunnel syndrome, *CVRR* Coefficient of variation in electrocardiographic R-R interval variability, *DPD* 3,3-diphosphono-1,2-propanodicarboxylic acid, *ECG* Electrocardiography, *ECHO* Echocardiography, *ECV* Extracellular volume, *FLC* Free light chain, *HMDP* Hydroxymethylene diphosphonate, *LV* Left ventricular, *LVWT* Left ventricular wall thickness, *MR* Magnetic resonance, *MRI* Magnetic resonance imaging, *NT-proBNP* N-terminal probrain natriuretic peptide, *PYP* Pyrophosphate, *TTR* Transthyretin

^a^Criteria for low voltage is present in only 25% of patients with ATTRwt; most patients, however, will have a low “voltage to mass” ratio

### Clinical suspicion of neural involvement: presenting symptoms and common misdiagnoses

The wide spectrum of clinical presentation and inaugural manifestations makes amyloid neuropathy a “chameleon-like” neuropathy; consequently, early diagnosis of ATTRv-PN can be challenging for general practitioners and specialists. The most common presenting symptoms of neurologic involvement include progressive sensory polyneuropathy, autonomic dysfunction (eg, chronic or alternating diarrhea and/or constipation, erectile dysfunction, and postural hypotension), pain in the hands or feet, and gait disorders [1, 14, 16, 65, 66]. Patients often present with other systemic signs, such as unexplained weight loss, cardiac symptoms (as already described), ocular manifestations, or renal abnormalities. Neurologic involvement frequently precedes the appearance of cardiac involvement by more than 5 years and often indicates that the disease is genetic in origin (ATTRv). CTS may be a particularly early sign for certain variants of ATTRv (eg, T60A) [66, 67].

Misdiagnoses of ATTR amyloidosis with neuropathy commonly include chronic inflammatory demyelinating polyradiculoneuropathy (CIDP), idiopathic axonal polyneuropathy, lumbar spinal stenosis, diabetic neuropathy, CTS, paraneoplastic neuropathy, monoclonal gammopathy–associated neuropathy, and, more rarely, motor neuropathy, inherited neuropathy, and amyotrophic lateral sclerosis (Table 2) [23, 65, 68–70].

### Clinical suspicion of gastrointestinal involvement: presenting symptoms and common misdiagnoses

The most common presenting symptoms of amyloid-related GI disturbances include chronic diarrhea or diarrhea alternating with constipation, unintentional weight loss often associated with early satiety, and a typical absence of abdominal pain [13, 20]. It is not uncommon for ATTRv to manifest first in the GI tract; therefore, the absence of peripheral neuropathy should not preclude clinical suspicion [71]. When patients present with nausea, vomiting, diarrhea, and weight loss, recognizing other impaired organs can be useful in leading to identification of a systemic disorder. Nephrotic-range proteinuria and cardiomyopathy are ancillary clues that the GI symptoms are amyloid in origin. Patients are often misdiagnosed with irritable bowel syndrome (IBS), unexplained malabsorption syndrome, protein-losing enteropathy secondary to ischemia, celiac disease, or infection. Absence of abdominal pain with a diagnosis of IBS may indicate amyloidosis (Table 2). Unfortunately, there are no specific findings for ATTR enteropathy on routine GI examination (ie, abdominal x-ray, computed tomography, colonoscopy/esophago-gastroduodenoscopy [OGD]) [72]; however, if a biopsy specimen from the GI tract contains the mucosal muscle layer and submucosa, amyloid can be detected with the use of Congo red staining. In addition, gastric retention observed during OGD and coexisting autonomic dysfunction should increase suspicion [73–75].

### Diagnosis

**Fig. 3:**
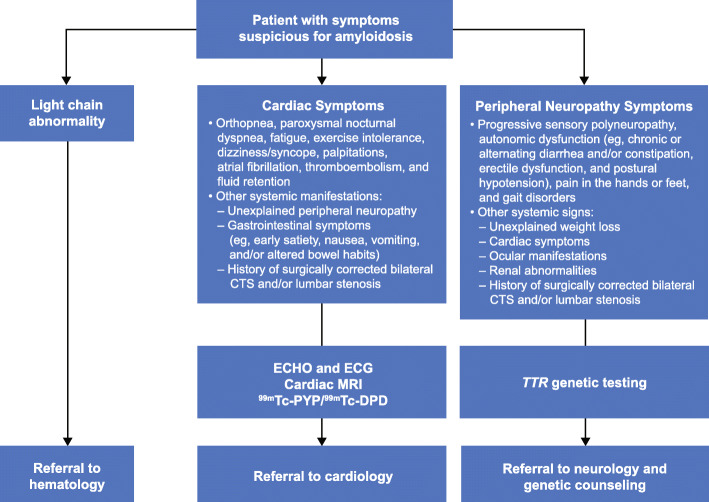
A general practitioner’s algorithm for increased suspicion and diagnosis of ATTR amyloidosis. Schematic of the recommended diagnostic approach for the general practitioner. ^99m^Tc-DPD, technetium-99 m-3,3-diphosphono-1,2 propanodicarboxylic acid; ^99m^Tc-PYP, technetium-99 m pyrophosphate; ATTR, transthyretin amyloid; MRI, magnetic resonance imaging; TTR, transthyretin

Although histologic documentation of amyloid remains the gold standard for diagnosis, a patient who exhibits any of the presenting signs or symptoms or who has received a diagnosis that appears inconsistent with the overall signs and symptoms should be referred to the appropriate specialist (Figs. 1, 3). Evaluations should include ECHO and/or CMR imaging to identify any thickening of the cardiac walls (as already described) [76]. Symptoms of progressive peripheral neuropathy and/or autonomic dysfunction should prompt a careful evaluation of family history, and a clinical history of CTS should be elicited [45]. Bone scintigraphy should be performed if serum/urine immunofixation and serum free light chain (FLC) levels are normal; in such cases, bone scintigraphy typically confirms or excludes a diagnosis of ATTR-CM [77, 78]. Bone scintigraphy documenting intense myocardial tracer uptake in the absence of any monoclonal protein in the plasma can be used to make a definitive diagnosis in the case of cardiac and mixed phenotype. If serum/urine immunofixation or FLC levels indicate the presence of a monoclonal immunoglobulin or light chain, a biopsy is required to confirm or exclude the diagnosis. Similarly, if bone scintigraphy is not available or myocardial uptake is mild (score 1), patients should be referred to an experienced center that offers bone scintigraphy or a symptomatic organ should be biopsied for evidence of amyloid and typed by mass spectroscopy or immunostaining [45]. It should be noted that while salivary gland biopsy is comparable to nerve biopsy to confirm a diagnosis, heart biopsy may be preferred in a patient with suspected cardiac involvement [79, 80]. Nevertheless, there are some potential pitfalls to this diagnostic approach:

- Tracer uptake can be sparse or absent in patients with definitive cardiomyopathy related to rare mutations (eg, *TTR* Phe64Leu)
- When PYP is used as the bone tracer, false-positive myocardial uptake (score 1) at planar images can occur owing to blood pool effect, such that single-photon emission computerized tomography (SPECT) imaging will be necessary
- Because grade 2–3 uptake may also be seen in AL amyloidosis, serum and urine immunofixation and FLC levels must be normal for ATTR-CM to be diagnosed using only bone scintigraphy
- Identification of a monoclonal protein is not diagnostic of AL amyloidosis. Monoclonal gammopathy of undetermined significance can coexist with ATTRwt, especially in elderly patients
- Interpretation of a biopsy specimen has certain challenges. Indeed, the sensitivity of non–cardiac tissue biopsy (and of abdominal fat biopsy in particular) in patients with ATTRwt is low

In cases of ATTRv with a neurologic phenotype (peripheral neuropathy) and no overt cardiac involvement, evaluations of blood glucose, serum B_12_ with metabolites (methylmalonic acid with or without homocysteine), and FLC and serum and urine immunofixation electrophoresis should be performed [81]. *TTR* gene sequencing should be performed in cases of progressive polyneuropathy of seemingly unknown cause accompanied by one of the following red flags: autonomic dysfunction or gastrointestinal disorders, bilateral carpal tunnel syndrome, unexplained weight loss [1, 82], early gait disability [66], or CIDP refractory to intravenous immunoglobulin therapy [1]. If one of the 130+ amyloidogenic variants is detected [83], ATTRv polyneuropathy should also be considered and patients referred to a center specializing in neuromuscular diseases.

Once a diagnosis of ATTR amyloidosis has been made, genetic testing by *TTR* gene sequencing should be performed to establish mutation status. Patients may be referred to an amyloidosis center for further evaluation and treatment. Genetic testing is recommended in all cases of suspected ATTR amyloidosis to aid in diagnosis and to identify whether the disease is hereditary. If the results are positive, family members may choose to see a genetic counselor, be tested themselves, and be monitored for the onset of symptomatic disease [49]. At this phase of the diagnostic workup, the main pitfall of immunohistochemistry is related to the suboptimal specificity of many commercially available antibodies, sometimes leading to simultaneous positivity for ATTR and kappa or lambda light chains. Mass spectrometry is generally able to detect the “leading protein” responsible for the tissue accumulation of amyloid deposits.

## Conclusions

ATTR amyloidosis, a life-threatening and underrecognized disease, was formerly considered untreatable; however, the recent availability of disease-modifying therapies has renewed efforts to increase awareness of the initial disease symptoms and the assessments that are available to confirm a diagnosis. A diagnostic algorithm is recommended for the general practitioner based on initial red flag symptoms and manifestations of cardiac or neurologic involvement, and the early use of genetic testing in patients with unexplained peripheral neuropathy should trigger referral to a multidisciplinary team at an expert center [1, 17, 83–87].

## Data Availability

All data generated and analyzed during this study are included in the cited published articles.

## Abbreviations

^99m^Tc-DPD: Technetium-99 m-3,3-diphosphono-1,2 propanodicarboxylic acid
^99m^Tc-PYP: Technetium-99 m pyrophosphate
ATTR: Transthyretin amyloid
ATTRv: ATTR variant
ATTR-CM: Transthyretin amyloid cardiomyopathy
ATTRv-CM: ATTRv-related cardiomyopathy
ATTRv-PN: ATTRv-related peripheral neuropathy
ATTRwt: Wild-type ATTR
ATTRwt-CM: ATTRwt-related cardiomyopathy
BNP: Brain natriuretic peptide
CIDP: Chronic inflammatory demyelinating polyradiculoneuropathy
CMR: Cardiovascular magnetic resonance
CT: Computed tomography
CTS: Carpal tunnel syndrome
CVRR: Coefficient of variation in electrocardiographic R-R interval variability
DPD: 3,3-diphosphono-1,2-propanodicarboxylic acid
ECG: Electrocardiography
ECV: Extracellular volume
ECHO: Echocardiographic
ER: Emergency room
FLC: Free light chain
GI: Gastrointestinal
HF: Heart failure
HFpEF: Heart failure with preserved ejection fraction
HMDP: Hydroxymethylene diphosphonate
IBS: Irritable bowel syndrome
IP: Inpatient admissions
LV: Left ventricle
LVWT: Left ventricular wall thickness
MR: Magnetic resonance
MRI: Magnetic resonance imaging
NT-proBNP: N-terminal probrain natriuretic peptide
OGD: Esophago-gastroduodenoscopy
OH: Orthostatic hypotension
OP: Outpatient services
PYP: Pyrophosphate
SF-36: 36-Item Short Form Health Survey
SNAP: Sensory nerve action potential
SPECT: Single-photon emission computerized tomography
TTR: Transthyretin
